# The Challenges of Educational Reintegration and the Psychosocial Wellbeing of Returnee Children: Evidence from Latvia

**DOI:** 10.1007/s12134-022-00960-3

**Published:** 2022-05-04

**Authors:** Daina Grosa, Russell King

**Affiliations:** 1 Institute of Philosophy and Sociology, University of Latvia, Riga, Latvia; 2 School of Global Studies, University of Sussex, Brighton, UK; 3 Department of Geography, University of Sussex, Brighton, UK; 4 Malmö Institute for Studies of Migration, Diversity and Welfare, Malmö University, Malmö, Sweden

**Keywords:** Return migration, Family migration, Children, Education, Wellbeing, Latvia

## Abstract

When emigrant families return-migrate to their homeland, what happens to their school-age children? What challenges do these children face when they switch to a different school system and language? This paper addresses these questions in the context of family return migration to Latvia, based on 40 in-depth interviews with children, their parents and key informants — teachers, school support staff and return-migration coordinators. We find that imaginings of a smooth reintegration into a parental homeland of extended family and friends may not be realised; instead, many children, particularly those of secondary and upper primary-school age, experience the move as a rupture in their lives. School may be fraught with unrealistic expectations on all sides, not helped by poor communication between parents, teachers and support staff. The lack of fluency in the Latvian language is seen by teachers as an obstacle, rather than something to be accepted and worked with. Most teachers are unfamiliar with children from different backgrounds and origins and need training in diversity, tolerance and differentiated learning. This will become increasingly necessary in a country like Latvia, with its ongoing high rates of international migration and return. Our findings show that the educational system and children’s experiences of schooling play a crucial role in returnee families’ overall reintegration. This raises the importance of return preparedness for the children, including language preparation and awareness of pedagogical and curriculum differences.

## Introduction

What happens to school-age children in migrant families when those families decide to return to their home countries? How do they face the challenge of integrating into a new environment and school system, with a different curriculum, different styles of teaching and, above all, lessons in a language in which they may not be fluent? What can schools in the parental home country do to help the ‘returnee’ children settle in?

This trio of questions highlights the salience of children in family decision-making about return migration. The geographical setting for the research is Latvia, which has witnessed large-scale emigration over the past two or three decades. Substantial return flows have been folded into the dynamics of Latvian migration, always, however, with an excess of emigrants over returnees. The recent history of Latvia is a geopolitical roller-coaster. Several decades as part of the Soviet Union came to an end with independence in 1991 and the long-awaited opportunity for people to emigrate. European Union membership in 2004 completed the geopolitical transition from communism to Western democracy and capitalism. Crucially, EU accession opened the gateway to free movement within Europe ^1^.

Our study of ‘returning’ Latvian children reflects a recognition of the previously overlooked role of children in migration, with even less attention paid to their situation in return migration (Grosa, 2022). If children were generally seen as collateral ‘luggage’ when families returned, this paper is part of a new strand of migration research which focuses on the wellbeing of children in the return-migration process. We offer a novel, multi-perspective approach to the psychosocial wellbeing of return-migrant children in their parents’ homeland by giving voice to three sets of actors — the children’s parents, the children themselves and key actors such as psychologists and teachers. Our key focus, however, is on the children and their educational reintegration and on what could be done to better prepare them for a transition to a new school system to which it is often challenging for them to adapt.

It is debatable whether the children in this study are true return migrants, since many of them were born abroad and therefore should be considered as migrating to a new country when their parents return. However, our sample also includes children born in Latvia and taken abroad by their parents at pre-school-age. The latter are, statistically, ‘true’ returnees whereas the former are not. For simplicity’s sake, when we refer to ‘returnee children’, we include both categories.

The paper unfolds as follows. First, we enlarge on the point made above concerning the overlooked role of children in return migration. The succeeding section introduces the conceptual framework, which relies on psychosocial wellbeing as its core idea. Next, we provide some background on Latvian migration, framed by economic factors set within a shifting geopolitical landscape. This is followed by an outline of the study’s methodology — 40 in-depth interviews, backed up by contextual reference to a large-N online questionnaire survey. The findings of the research are presented under several heads: general insights from the questionnaire survey, motivations for return on the part of the parents, preparation for the return, children’s school experiences after return and communication between family and school. The conclusion sums up key findings and then turns to issues of policy for a better educational integration of returnee children in the future.

### Children in Migration and Return and the Challenge of their Educational (Re)Integration

Apart from the humanitarian and policy-focused concentration on unaccompanied minors, the far more numerous children who move transnationally as part of family migration have been less studied by migration scholars (Grosa, 2022). Traditionally seen as part of the ‘baggage’ of families’ migration, recent years have seen a powerful call for more attention to be paid to the migration experiences of children. Key milestones in this literature have been several important, mainly edited, books devoted to children’s role in migration (Assmuth et al., 2018; Coe et al., 2011; Ensor & Gozdziak 2010; Ní Laoire et al., 2011; Parreñas, 2005), two special issues of the *Journal of Ethnic and Migration Studies* dedicated to this theme (Gardner & Mand, 2012; White et al., 2011) and some landmark papers (e.g. Bushin, 2009; Dobson, 2009; Orellana et al., 2001).

Despite this growing attention to children as migrants, the role of children in *return* migration has been routinely overlooked. Previous studies have looked at the influence of children on family decision-making about return (Dustmann, 2003), the problems suffered by children in refugee families forced to return (Cornish et al., 1999; Zevulun et al., 2017) and children’s home-making practices in the context of highly skilled returning families (Hatfield, 2010). As signalled by the three questions in the “Introduction”, our interest is on school-age children and their experience of the new school setting after their return to Latvia. On this, very few studies exist, located mainly in two contrasting geographical contexts. Research on Albanian returnee children (see Vathi & Duci, 2016; Vathi & King, 2021; Vathi et al., 2016) has some parallels with our Latvian study, as both involve return migration to European post-socialist countries. Second, papers on the Mexican-American situation recognise both voluntary return migration and, in the case of ‘illegal’ status or criminal activity, forced repatriation (see Jensen & Jacobo-Suárez, 2019; Medina & Menjívar, 2015; Zúñiga & Hamann, 2015).

What can be learnt from this extant literature? Vathi and King (2020) found a process of ‘double othering’, mirrored between the host-country setting (Greece) and the parental homeland (Albania). As the offspring of Albanian immigrants (a stigmatised group in Greece), the children were ‘othered’ in their Greek schools. Yet, when they were taken back to Albania because of the unemployment of their parents following the Greek economic crisis, they were again othered in Albanian schools due to their ‘Greekness’ and inability to speak Albanian fluently. The returnee children, for their part, reciprocally othered the local Albanian children as being, in their view, rough and ‘uncivilised’.

Other research in the same complex geopolitical realm of South-East Europe sheds light on different, yet complementary, aspects of children’s ‘return’. A study on migrant and asylum-seeker children returned to Kosovo found that those who had the most problematic reintegration were the Roma children of ‘failed’ asylum-seekers, whose marginal status in society was further enhanced by their forced return (Zevulun et al., 2017). In the different geopolitical context of the ethnic repatriation of diaspora Greeks from the former Soviet Union to Greece, Kolaitis et al. (2003) found no statistically significant difference between the diasporic children and a control group of ‘native’ children in terms of psychosocial wellbeing, although there were significant differences in school academic performance — the repatriate group being below par, especially in language-related areas.

Albeit on a much larger geographical and numerical scale, Mexican-American children returning to Mexico — a flow which has increased considerably since 2005 (Medina & Menjívar, 2015) — face serious problems in their schooling. They struggle to integrate into Mexican schools, resulting in academic underachievement and high dropout rates (Jensen & Jacobo-Suárez, 2019). Many face legal and bureaucratic challenges over proof of citizenship and rights to access schooling, as well as barriers to receiving credit for studies completed in the USA (Medina & Menjívar, 2015). Mexican teachers fail to understand their particular needs regarding fluency in Spanish and adaptation to different cultural norms and pedagogic practice (Jensen & Jacobo-Suárez, 2019).

Finally, a very different perspective emerges from Ní Laoire’s (2011) research in the west of Ireland. Returnee children from Britain and the USA do not face a language barrier — except due to their different accents, for which they are often teased. Aside from this, the returnee children and their parents generally welcomed the greater freedom, space and feeling of safety they enjoyed in Irish rural and small-town schools, in sharp contrast to their experiences in large urban schools in Britain and the USA prior to their return.

### Psychosocial Wellbeing and the ‘Return’ of Migrant Children

In this paper, we adopt a psychosocial lens to youth/child wellbeing in the context of return migration. As an aspirational ideal state, human wellbeing has become a catchword in contemporary society (McLeod & Wright, 2015). Writing in a migration context, Wright (2012) distinguishes between so-called objective wellbeing, based on measurable indicators such as material wealth, physical health and ‘subjective wellbeing’, which includes more emotional, relational and perceptual aspects. The subjective approach is closer to psychosocial wellbeing, which Vathi (2017: 5) defines as ‘a person-centred concept that emphasises the value of interactions, social and emotional consonance, and individual experience’.

We focus on the psychosocial wellbeing of returnee pupils in Latvian schools. Schools are widely recognised as the key setting for children’s experiences of reintegration even if, as we shall see, they may not be structured in the best way to receive returnee children coming from a different educational system. Having said that, we recognise, with Chapman (2015), that school children’s wellbeing is not a neutral concept, given the implied value-judgements in what is to be measured. A holistic approach is arguably the most appropriate, combining objective measures of material life, friendship networks and academic performance with the more subjective dimensions of life satisfaction, happiness, self-realisation and lack of stress.

Vathi and King’s (2017) edited collection offers several case-studies of the interface between psychosocial wellbeing and return migration, although only two chapters deal with children/youth in education. Gońda (2017) looks at coping strategies and psychosocial outcomes for ethnic Polish students from the Polish diaspora in the former Soviet Union who enrolled in Polish universities in their late teenage years: a form of ‘roots migration’ via the education route. Despite their emotional links and patriotism towards their ancestral homeland, they were treated as ‘strangers from the East’ and discriminated against in various ways, resulting in disillusionment, alienation and depression for many of them. In the second contribution, Lulle (2017) studies Latvian youth returnees through a biographical dialogic lens and includes a couple of case-histories which resonate strongly with our research — the embarrassment of having a foreign accent, the shame of being put with younger children because of language deficiency and psychosocial withdrawal as a result.

Our paper, based on a larger scale of empirical research, contributes to the wider literature on returnee children. In addition, it offers a practical slant on how parents can potentially avoid the negative pitfalls of return through preparation, a positive attitude and constructive communication practices. It also outlines government support measures showing the state’s efforts to ease the integration of returnees.

### Latvia: Migration and Geopolitics

One of the three Baltic states that were part of the Soviet Union until 1991 (the others were Estonia and Lithuania), Latvia moved swiftly towards Western Europe after the break-up of the Soviet empire. Over the past 30 years, and especially since EU accession in 2004, Latvia has witnessed a high level of emigration to richer European countries (primarily the UK, Ireland, Germany and the Nordic countries) and beyond to North America and Australia. Estimating the scale of this migration is difficult because of the free-movement space of the EU and the ‘churning’ effects of seasonal and return migration. Statistics indicate a net migration loss of 405,000 during 1991–2013 (Krišjāne et al., 2016). According to data from the Central Statistical Bureau of Latvia (2021), the country’s population fell by 28%, from 2,668,000 in 1990 to 1,910,000 in 2020, due to a combination of high net emigration and low birth rate — a worrying demographic trend, which makes return migration all the more important for the long-term future of the Latvian population.

If emigration decisions were generally clear-cut and dictated by economic factors, the return decision is inherently more complex and shaped by a variety of factors, many of them non-economic (Gmelch, 1980). Online survey data with 2565 respondents collected by Krišjāne et al. (2016) on return intentions (not actual returns) showed that those more likely to plan return were males, families with children and people with higher education. Many were influenced by the social attractiveness of the country compared to how it was when they left. However, returning parents with younger or school-age children expressed worries about the availability of places in local schools and pre-schools and whether schools have support measures in place to integrate returnee children — concerns which are enshrined in the questions at the start of this paper.

Beyond the school environment, there are other factors which contribute to the hesitation of potential returnees to make the move. Due to the scale of the emigration combined with nationalistic sentiments, an ‘us and them’ mentality developed in public discourse, whereby those who left were seen as ‘traitors’ and a different category of Latvians (Lulle 2007). An additional confounding factor is the survival of a post-Soviet mentality, with some ingrained norms and behaviours that are slow to change in response to Latvia’s geopolitical reorientation towards the European ‘West’. According to Municio-Larsson (2012), family-life repertoires are different in Latvia compared to countries in the West.

Another enduring geopolitical legacy of the Soviet era was the in-migration of an estimated 700,000 Russians, constituting roughly one-third of the Latvian population. A complex relationship still exists with ethnic Russians in Latvia, a sizeable proportion of whom speak only Russian in their daily lives. This, coupled with a general mistrust of immigrants from outside the European Union, contributes to a low public tolerance towards diversity, multiculturalism and the inclusion of ‘others’ in Latvian society. Using Eurostat data, Zubikova (2020) found that Latvia had the lowest level of immigrant integration (along with Slovenia) of all the 10 states which joined the EU in 2004. Although return migrants do not fall into the category of immigrants *per se*, they constitute a growing inflow entering the country. To the extent that they have different life experiences and potentially altered cultural characteristics, they are regarded as partial ‘outsiders’, even if their return is prompted by a sense of national belonging (Lulle, 2017).

Still, following the example of other countries in promoting and supporting the return of diaspora members to their homeland (Šūpule et al., 2016), various support measures have been implemented by the Latvian government to counteract an ageing Latvian population, the consequent high age-dependency ratio and brain drain (Hazans 2015). These are outlined in the Diaspora Law, 2018, among the aims of which is the inclusion of support measures for returning nationals in the education system. Promoting the return of Latvian nationals is also included in a 2021 regulation of the Latvian government, with support for return seen to be a significant investment in the future development of the country (Cabinet of Ministers Regulations, 2021, No. 33).

## Methodology

Material for this paper was collected as part of a wider study on Latvian migrants abroad and returnees to Latvia ^2^. The study comprised a quantitative survey (see Mieriņa et al., 2020a) and, more importantly for this article, a qualitative part consisting mainly of in-depth interviews. Interviewees were recruited via targeted social-media groups, supplemented by snowballing. They included returnee parents of school-age children, the children and young people themselves and teachers and other key informants — such as support staff and return-migration coordinators — with experience and knowledge of the issues facing returning families. The total number of 40 persons interviewed represented 15 families made up of 20 parents and 11 children, plus key informants. Of the 20 parents, 17 were mothers and, of the children, six were girls and five boys. The children ranged in age from 8 to 18 at the time of interview. Four were born abroad and seven were born in Latvia and taken abroad at pre-school age. The predominance of mothers in parent interviews was due to their much more positive responses to requests for interview and because, on the whole, mothers are more likely to be directly involved in their children’s school arrangements and social lives. The families had returned from various parts of the world including traditional overseas ‘settler’ countries (Canada, USA, Australia) and several European countries of more-recent Latvian emigration (Germany, Norway, the UK, Ireland).

Children were approached following interviews with one (or both) of their parents and upon the latter’s recommendation and approval; informed consent was also elicited from the children themselves. They were interviewed either in their homes (with parents in the next room) or (for older children) in public spaces such as libraries or cafés. Some interviews had to be via Skype, especially after the onset of the coronavirus pandemic in early 2020. Ethical safeguards for the children were strictly adhered to, with care and discretion exercised during interviewing, veering away from topics that might cause distress. All participants were given pseudonyms.

The interviews were semi-structured and, although initiated and guided by the interviewer (the first author), themes that the interviewees placed emphasis on and spent more time discussing were encouraged in order to highlight what *they* thought was important. All the interviews were recorded and transcribed. Texts were thematically analysed using the NVivo qualitative-analysis programme. The average interview length was approximately 1 h, with a range from 20 min to 1.5 h.

With variable emphases for parents and children, the interview themes covered feelings about returning to Latvia, reasons for the return, how families prepared for it, concerns about moving into the new (or perhaps familiar) living environment and worries about the children’s schooling. Participants were questioned on the language maintenance and proficiency of the children, their socialisation and performance at school, the support measures available to aid educational integration and schools’ expectations of their pupils and parents. Comparisons between the schooling experience in the two countries, Latvia and abroad, were encouraged. In their interviews, parents were asked to look at the return move through the eyes of their children, structuring their narratives around the experience, as they saw it, of the child. Teachers and other key informants were also asked to share their perspectives on how returnee children fared at school, not just academically but also socially and emotionally.

Some degree of triangulation of results was achieved via an integration of voices from three experiential viewpoints — parents, children and key informants. However, we were also prepared for the reverse to happen — as in the case described by Assmuth and Siim (2018) of Estonian children in Finland — where, in interviews, the parents wanted to imply that their children had settled relatively smoothly into the school system, yet the children articulated an opposite experience.

## Brief Perspectives from the Quantitative Survey

Although the bulk of the empirical evidence presented in this paper derives from the interviews, some general findings from the online survey are useful to set the scene. The survey covered 7700 respondents, one-third of whom were returnees to Latvia. Respondents with children (*n* = 2477) who *still lived abroad* were clear about which factors would hinder their children’s integration into the Latvian school system. The most commonly expressed worry was the lack of proficiency in the Latvian language (nominated by 73% of the respondents). Other factors frequently identified were the different teaching methods in Latvia (59% of respondents), teachers’ unempathetic attitudes towards newcomer pupils (46 per cent), other children’s attitudes (41 per cent) and differences in curriculum content (35%).

When this question was put to *returnees* with children (*n* = 351), a different picture emerged: 41% stated that nothing hindered their children’s adaptation, while only 26% mentioned that children’s proficiency in the Latvian language and also the attitude of teachers towards newcomers posed a challenge. Differences in curriculum content (16%) and the attitude of classmates to newcomers (13%) were recognised as challenges by smaller shares of the returnee subsample. An overall 53% of returnees replied that integration into the education system in Latvia has been either very easy or easy. These responses show that challenges anticipated on return were not as great in reality, as the satisfaction rate of returnees with their children’s integration into the education system was overall quite positive.

Respondents were able to go into more detail in answers to open questions in the survey. Compared to teachers in the various host countries, Latvian teachers were thought to have an authoritarian approach. Many respondents highlighted a lack of support for helping newcomer children to adapt and integrate. This was often put down to the perception that most teachers in Latvia are quite old, with their formative training and early teaching experience under the Soviet system.

The survey findings are clear and all the more impressive given the large sample size. However, it is in the nature of online surveys not to be fully representative. Moreover, such surveys involve quite short questions and a limited range of responses. For a more-nuanced and multi-vocal account, we turn to the interview narratives.

### Motivations for Return and Consequent Implications

Most studies agree that, aside from forced return, return motivations are inherently more complex than the reasons behind the preceding decision to emigrate (see reviews by Gmelch, 1980; King, 2000). The Latvian data, both from the questionnaire survey and the interview narratives, support this generalisation. Latvian emigration has been economically driven — to seek work or a better job and a higher and more secure income or, in a few cases, to study abroad. According to the interviews with parents, return was motivated by a variety of factors: completion of the migration plan (for instance, successful repayment of debts, money saved for home or investment in a business) or, conversely, a sense of disappointment at the outcome of migration (low wages, high cost of living, cramped housing, difficult integration etc.). Nostalgia and homesickness also played a role for some returnees, although sometimes these labels were probably used to hide deeper-seated or more-shameful situations, such as personal difficulties or a failed relationship.

Regarding the children, one motivation which stood out in some interviews and survey responses was the desire to return once the children approached school age. Roberts, who returned with his wife and two pre-school daughters from Ireland, shares his story:

I became used to life in Ireland; for me, everything was fine. I was happy… and prepared to stay but the children were the reason we returned. They had to start school and we had to decide where we wanted out children educated. At that moment we started to consider coming back to Latvia; we started making a list of the pros and cons… We didn’t want our children to remain in Ireland and start attending school because, if we were to return to Latvia later, if we had to take them out of their school… What I am trying to say is… we wouldn’t go, if our children were attending school [in Ireland]… So, in a way, we caught the last train…

In this quote Roberts describes, albeit in a somewhat hesitant way, the classic dilemma of migrant families when their children reach school-age. Staying in the host country means the children start their educational career there, in the host-country language, following the schooling system and acquiring a school-based friendship network. Roberts is anticipating the issues faced by parents — and particularly their children — if the return takes place when the latter are older and already in school. Note, too, how Roberts is silent on the views of his (admittedly very young) children. He declares that his children ‘were the reason we returned’ but does not say how the children felt about this.

At this point and to foreground some of the evidence presented later about the problems of children switching school systems, we quote extensively from a key-informant interview with a psychologist in Latvia who has considerable experience of working with return-migrant families.

Some of them [parents] return because they have become disillusioned. Or life hasn’t turned out the way they wanted… They return simply because this is their home and, for example, their parents are here and they have a place to stay. Then there is the question of the loss that the child experiences… Everything was going fine for them: they were at school, had friends, had settled in well. They are pulled out and are then placed in a completely different system…

I get to witness that the child’s psychological wellbeing … comes to the fore. We usually like to think that everything comes back to language but schools often don’t know this… They also don’t take into account the child’s context. Because return often takes place against a rather negative backdrop. For instance, the parents’ hopes have been disillusioned… they had expectations that life abroad would be something else… Or, whilst they were abroad, the parents split up.

Admittedly, psychologists will, by the nature of their work, be seeing the more-distressed returnee parents and their children. Their insights, however, do highlight the fact that return scenarios are not always simple — parents make decisions based on their interpretation of a particular life situation and uprooted children can become unwilling ‘pawns’ in a return move.

### Teachers Admit that Assistance is Inadequate

For returnee children with very limited or no Latvian language skills, entering the government school system can be traumatic. Although government regulations stipulate that returnee children may be supported with additional individual tuition by teachers after school hours for a period of 1–3 years (Cabinet of Ministers Regulations 2015, No. 591), Tamāra, a Latvian language and literature teacher from a prestigious government high school, admits this is not sufficient. Her attempts to help a returnee child in Grade 8 proved to be a challenge for both teacher and student.

Tamāra: No, unfortunately for those who have none [Latvian language skills], there is no specific offer of assistance. We did have the option of offering an extra few hours a week, additional to standard consultations [available to all children], which are in addition to the standard lessons…

Interviewer: Is this even achievable?

Tamāra: It was very, very complicated, as these pupils must attend all standard lessons...the Grade 8 list of subjects is already extensive, so then [these extra consultation classes] are offered late in the afternoon...so with this girl I tried to work with her during Home Economics classes...any free time that was available...I tried.

Interviewer: What could you even offer her, if there is no programme for beginners...what did you actually do [in these extra lessons]?

Tamāra: It might seem extremely nonsensical and absurd but because the pupil would not be given any concessions – none whatsover – then my task was to help [her] learn the curriculum planned for that year, meant for all pupils in that grade...and for the pupil to learn the basics – they need to do this in their free time, outside school hours…you reach an impasse.

This particular situation ended with the pupil, in Tamāra’s opinion, being very confused and frustrated — the curriculum seeming to be ‘something from outer space’ — and her pupil ended up returning with her family to the USA.

From this experience, we can deduce that having a teaching assistant at school in the initial period would help with recent returnee children’s integration into the education system (Hazans 2016). Teaching assistants are also mentioned in government support measures for returnees, but they are subject to municipal funding, which is often insufficient. Individual lesson plans are another school support measure, yet they are not always utilised.

The Latvian government does offer schools additional funding via a programme entitled ‘PuMPuRS’ — to reduce the risk of youth leaving school prematurely (Latvian Language Agency 2021). Schools can apply for support for returnee youth (in the form of additional support staff) via this programme. Despite these support measures, there is still no option for returnee children to learn and improve their Latvian, as a beginner or intermediate learner, within the education system.

### Preparation is Important: the Role of ‘Diaspora Schools’

Preparation for return migration may seem an easier task than moving abroad because return signifies going back to something that is known. In his two papers on re-thinking return migration, Cassarino (2004, 2008) explores the different levels of preparedness exhibited by returnees, both at an individual psychological level and from the point of view of the society of the country of origin and that society’s capacity to ‘welcome’ returnees into the labour market. Returnees who have developed a more transnational lifestyle and mindset, including regular visits to the home country whilst they were abroad, are more likely to have a better insight into the reality of life in the homeland and therefore be able to enact a successful return.

For children, however, it is a different story. Their human capital is not yet identifiable in terms of its benefit to the labour market, and their feelings regarding (re)integration cannot be categorised in the same terms as those of their parents. For some children, especially those in overseas settler countries, the move to the country of origin may be their first visit there and the first move away from familiar territory. This was the case for Vilis, aged 18 when interviewed, who moved from Australia to Latvia aged 6, when his parents returned.

Actually, I didn’t even know what it meant to move, I didn’t know that my relationships with my classmates would end, that my life would change 180 degrees, I didn’t know anything about this; it all seemed to me like one big adventure… I only had this fantasy Latvia in my mind… I don’t think, at that age, I knew what it all meant…

A young child probably has no way of cognitively appreciating such a big move before it happens; their only points of reference are their parents’ portrayal of what awaits them and their memories of any holiday visits they may have made.

The challenge for parents is twofold: firstly, how to include children in the return decision-making process and, secondly, to convince them that they will benefit from and enjoy the life-changing return move. For one family in the sample, returning after 7 years in England, the goal had always been to return when sufficient funds had been saved to build a family home in Latvia. Both the children had started primary school in England, the youngest in Grade 1 and the oldest, who had been born in Latvia, Grade 6. For this family, preparing their children for the move meant making the change in lifestyle the motivation. The family had been living in a rather crowded flat in England, and the alternative presented to the children by their parents was the chance to have their own, much larger, living space in Latvia, with their cousins living nearby and the promise of being able to keep a pet. As a result of interviewing different members of the family, it was possible to ascertain that, although there were some teething problems with leaving their friends behind in England, the move went quite smoothly, also helped by the 11-year-old being able to continue to play online computer games with his English friends.

Longer-term preparation can take the form of language maintenance — both using the language at home and taking a more academic approach. Improving the language via distance education and home learning with textbooks is a challenging regime demanding discipline and commitment, especially if the aim is to achieve a level that is expected in Latvian schools. Another option is to attend a ‘diaspora language’ school, usually at weekends. Based on experience amongst the Latvian community in Australia, Grosa (2015) found that attending a weekend heritage-language school was valued as an opportunity to meet other co-ethnics and learn about the culture of the home country; however, advanced language learning was not achieved. This view was confirmed by Ritma, a returnee mother with three children who had lived in the USA for 5 years:

Diaspora schools… don’t prepare one for return migration. Well, hardly at all… It depends on what we are talking about, and also on the level [of language teaching]… as each child is at a different level… A [diaspora] school can create in the child a sense of belonging to Latvia… but for them to graduate from the school and be able to go [and be ready]… for the Latvian education system – No.

In other words, a diaspora school is not a panacea for teaching the Latvian language, and the onus is on the parents to keep the language alive at home and maintain regular contact with Latvia. Diaspora schools are good for socialising with other diaspora Latvians and can give the children a sense that they are not the only ones who speak Latvian at home (Mieriņa et al., 2020b).

Other studies of so-called ‘second-generation returnees’ also reveal mixed, often negative memories of the usefulness of language schools in the host country. In Christou and King’s (2014) account of the Greek second generation growing up in Germany and the USA, there is frequent reference to ‘Greek schools’ in the host country, which were often seen as an extra burden over and above normal schooling. However, for those who had relocated as adults to their ethnic homeland, the earlier experiences of learning more about Greek language and culture were appraised as generally useful. Somewhat in contrast, young second-generation Turks in Germany recalled with horror the Turkish language and history classes taught as an adjunct to their German schooling (King and Kılınç 2013). The teachers, sent over from Turkey, were remembered as authoritarian bullies, and the emphasis was on military-style rote learning, in stark contrast to the much more liberal and pupil-centred German pedagogic system.

### Comparing and Contrasting School and Life Experiences

It is natural for returnee families to compare and contrast their lives in the host country with life back in Latvia. For children, it is the school experience which is the most pertinent. Fifteen-year-old Ilona spent a semester in a Latvian school, after which the family returned to Australia.

In Australia we had an assessment planner and I knew when my next exam was coming up… [for instance] in week 8 we have exams and you know that you will have them… In Latvia I never knew when the exams or assessment tests were coming up and there seemed to be so many of them.

In this case, with a pupil moving between two dissonant assessment cultures — one planned and pre-announced and the other less organised yet with more frequent exams — the student became stressed because of the challenge of juggling assessment deadlines and out-of-school activities.

Other interviewees commented on the different ‘atmosphere’ in the two school systems, including the behaviour of children in class. This was a concern expressed by many returnee parents and their children. Here, we take the example of Sigita, mother of 12-year-old Dainis. The family had returned from 10 years in Norway.

In his class [in Latvia], the children are rougher, less considerate, less polite, less tolerant. They don’t listen to what the teacher says. The teachers can’t cope with them; the children have no respect for the teacher… A teacher might be explaining something, and the children just shout during the lesson… one of them might constantly say ‘Miss, can I eat a chocolate?’; ‘Miss, may I eat a chocolate?’ So, the teacher has problems disciplining the children… That’s something that Dainis isn’t used to and it is difficult to acclimatise to because it is annoying. It wasn’t like that in his class [in Norway].

Sigita went on to acknowledge that disruptiveness can vary from school to school and even from one class to another in the same school, dependent on the individual teacher. It is certainly not restricted to return-migrant scenarios. Yet, these are issues of wide concern for returnee parents and children, as the earlier summary of questionnaire responses indicated. Such issues are found in other return-migrant settings too. Studies of Albanian children returning with their families from Greece in the aftermath of the severe Greek financial crisis found similar results. Local Albanian school children were experienced as physically rough — with frequent pushing and shoving — and prone to swearing and misbehaving (Cena et al., 2018; Vathi & King 2021)

Later in her interview, Sigita mentioned an incident where her son’s new classmates played a trick on him, resulting in Dainis missing an athletics event in which he was supposed to take part. Although practical jokes, teasing and bullying can happen in any school environment, the difference is the way it is tackled by the responsible authorities. Latvia has a poor track record in this field. According to Gobiņa et al. (2008), the practice of bullying is particularly widespread in Latvian and Lithuanian schools compared to other countries. Bullying is associated with poor subjective wellbeing and low life satisfaction (in both bully and victim). Returnee children are liable to being bullied because of the various markers that make them ‘stand out’ — their accents, lack of proficiency in the relevant language and its local slang and their different mannerisms, interests and cultural norms.

Returning to the case of Vilis, whom we quoted from earlier, his narrative is particularly revealing as he was able to look back on more than a decade of school experience in Latvia since his family’s return from Australia. In this quote, he shares his experience of being bullied at primary school.

I think in the first year [in Latvia]… I didn’t understand much of what was going on [with regard to bullying]. But then slowly I started to feel more intensely that I was different … that I wasn’t accepted… A number of little groups formed and I wasn’t in any of them and, rather quickly, there was some kind of discrimination against me. They started playing all sorts of nasty games… The teachers knew about this and occasionally would try to combat it… but the pranks still continued… As I look back on it now, I was very anxious and stressed, I couldn’t cope with my emotions…

Later, Vilis changed school and had a better experience. Looking back, he says he bears no grudge against his tormentors. As a young adult now, he feels his age, resilience and life experience have helped him to turn this negative experience into a positive one.

### Communication Between Family and School

Some of the more intangible obstacles to returnee pupils’ successful integration into the Latvian school system can be put down to a lack of communication between school and parents. Often, there had been a better culture of teacher–parent contact in the host country — including the frequency and style of meetings — and this contrast became a source of frustration for the parents and children involved. In the following interview extract, Ritma, a returnee mother of three school-age children, shares the experience of her son, who had his first encounter with a Latvian school in Grade 4. The example is perhaps banal but not untypical.

I went to school 30 years ago and I don’t remember how many squares from the left [your writing needs to be started in a squared exercise book], how many from the right… from the top, from the bottom… and when I asked the teacher where this was explained, she shrugged her shoulders. And I asked the head of teaching, and she too shrugs her shoulders. It seems that ‘everyone knows’, but what does that mean, everyone knows? We had a running joke that there is a special rule book just for our family floating round the school…

This may seem trivial but, precisely because of this, teachers take it for granted that all pupils know the rule from earlier years — but when they are asked to explain, they are stumped. The ironic situation here is that the family in question is not outsiders *per se*, yet because their son had not started school in Latvia, they did not know the minutiae of the ‘house rules’.

Government schools in Latvia are not renowned for good communication with parents, especially in bigger schools with large class sizes. It is the norm that individual contact with parents only takes place if there is a problem that needs solving. However, smaller state schools — for instance in more-provincial areas — and private schools are lauded for their more individual approach. For this reason, returnee families often choose these types of school over the larger, more prestigious ones, where often the child can become ‘invisible’, and gaps in knowledge can easily be missed. Vilis switched schools from a prestigious government school in the centre of a major town to an ‘alternative’ school an hour’s bus ride away:

I would say that the teachers [in the ‘alternative’ school] were much more prepared to start up a conversation, they have a warmer relationship with the children… the teachers were much calmer and happier… In my previous school, a lot of emphasis was placed on grades, on how well you were doing [academically]… whereas in this [the second] school, it did happen too, but to a lesser degree… In the morning we would spend 20 minutes to half an hour... the teacher would keep us updated with what was happening, we could ask them about things… I would say that the connection between the teacher and the class was much closer…

The above are just two instances from many in the interview data. A wider issue concerns the regulatory support mechanisms that are in place in government schools in Latvia. Theoretically, extra classes are available before or after school for language teaching or other specialist help, as is the option of having an individual learning plan set up. Teaching assistants can provide extra support in the classroom, as can school specialists such as an educational psychologist or speech therapist. A special grant scheme is available to schools to counter dropping out, in which returnee children with language support needs are included. A broader question, however, concerns the actual implementation of all these support measures, given that funding for them is often lacking and teachers do not have the differentiated teaching skills to integrate returnees in the classroom. The experience of Daila, who returned with her family, including her 8-year-old son, after spending 4 years in Canada, is instructive here; she enrolled her son in a private school to help him ease any difficulties settling in.

What options does one have in a government school, if a child arrives older than Grade 2 or 3? They are placed in a class with 30 children and no assistant… Theoretically, schools should provide this support, but in reality it’s not there. [At the private school that he attends in Latvia], the teacher then sits with Mārtiņš and he has individual tuition. They call it ‘superlearning’.

A lack of academic and psychological support in the initial weeks and months of settling in can have a demoralising effect on a returnee child’s long-term psychosocial wellbeing. If such children constantly achieve low grades and support for improving them is missing, then the motivation to learn can disappear altogether. The 2019 diaspora survey respondents, in answers to open questions, made several suggestions, including special allowances for returnee pupils in exams and tests and monitoring newcomer pupils carefully to ensure that they are fitting in (Mieriņa et al., 2020a). These suggestions are in conformity with recommendations made in other geographical contexts for returning migrant pupils. For instance, regarding Mexican-American pupils ‘returning’ from the USA, Jensen and Jacobo-Suárez (2019) advocate for school and district leaders in Mexico to organise basic language support so that these ‘transnational’ students can access the curriculum. These authors also suggest that interim assessments be taken in English and that there should be the use of non-verbal aids (pictures, demonstrations, hands-on activities etc.) to complement formal instruction.

## Conclusion and a Look to the Future

Although the online survey results show that some challenges anticipated by parents (such as language proficiency and the attitude of teachers to newcomers) are not as serious in reality, with returnees reporting more satisfaction, both the qualitative and quantitative findings show that there are still various aspects of children’s return that negatively affect their psychosocial wellbeing.

The main contribution of this paper has been to focus on the key role of schools in relation to both the preparedness of the children for ‘return’ and their reintegration. Policy-wise, tackling these two facets in conjunction, before and subsequent to the return, would surely contribute to a smoother transition for the returnee children and thus enhance their psychosocial wellbeing. Our evidence has revealed that there are numerous areas within the school setting where there is a dissonance between what has been familiar and the norm in the host country and the experience of the children in Latvia. Newly returned families inevitably compared the type of schooling prevalent in the host country with the Latvian system, which still has elements of rigidity and hierarchized learning that attest to the legacy of its Soviet past.

School systems, as experienced in different settings by migrant and returnee children, are partly reflective of histories of political and educational ideology. Children’s experiences of schooling are impacted on by these legacies, as well as by the shifting geopolitics and economics of migration to which their parents respond. Family migration and return decisions are shaped by all these factors in various combinations, yet children, who frequently do not have a say in these decisions, are often the most fundamentally affected over the longer term.

The challenge for returnee parents and children is to keep an open mind and not be overly critical of cultural approaches in education that may contrast with the norm in the host country. Supportive parents may spot differences, yet also see the positives in the new family-life situation and, if this observation is successfully transmitted to their children, this can help with the process of settling in. A positive mindset demonstrated by parents can help to calm distressed children. Children who lag behind because of curriculum differences or their lack of language fluency or who suffer from school bullying or an unsympathetic attitude from their teachers, are at risk of psychosocial distress. If parents are willing to act on their children’s behalf by speaking to teachers to tackle the problem jointly or by seeking out other forms of help, children will feel supported because of parental involvement in resolving issues. This in itself can mitigate any stress that may have arisen.

Ultimately, the success of the project of school reintegration for returnee children depends on their preparedness for the challenges they face (cf. Cassarino 2008). In the context of this research, preparedness breaks down into three key elements: (i) more serious attention to language competency, especially prior to return but ongoing thereafter, (ii) children’s cognitive geographies — their knowledge and perceptions of the homeland before they are taken there and (iii) parents’ involvement of the children in the return decision-making process and their engagement in presenting return to the children in a favourable light.

What can be done concretely to improve the situation for returnee children? Prior to return, weekend heritage-language or diaspora schools as well as diaspora summer camps (held in both the diaspora and Latvia) are considered effective places for strengthening cultural identity and helping with language maintenance but their role does not extend to preparation for return to the country of origin. Various forms of online tuition with teachers from Latvia are another option which has proven beneficial for some returnee pupils.

However, there is currently still a lack of a systematic approach to pupils who enter the government education system with little or no Latvian language expertise, nor is there a transition period during the initial months allowing grading concessions. Both of these strict measures can contribute to psychosocial stress in returnee pupils, particularly at high-school level, where it is difficult to keep up with the curriculum if the course content is unfamiliar and the pupil lacks proficiency in Latvian.

A more promising initiative for the longer term is the *Skola 2030* (‘School 2030’) programme, launched in the Latvian school year 2020–2021. This is a competency-based approach to teaching which, in addition to the standard mastery of skills, ability and knowledge, includes components on socio-emotional learning and inclusive education. Teaching resources have been developed and utilised by teachers to promote socio-emotional skills and good mental health in children. This not only has an effect at the individual level but also improves the school climate and creates an inclusive environment. The needs of return-migrant children, especially for additional language teaching, are explicitly recognised under the ‘special education’ category. In 2021, an online handbook was launched for return-migrant parents and also for teachers of returnee children, to help them to understand the challenges that such children may face.

Meanwhile, students who are currently studying pedagogy in Latvian universities are also taught socio-emotional learning and inclusive education, which includes returnees, as part of their teacher training. These newly graduated teachers will have acquired the knowledge and skills to implement the new *Skola 2030* programme, while existing teachers are offered professional development courses under the scheme. If the transition to the new approach is successful, then some of the problems of returnee children’s adaptation will be attenuated. The *Skola 2030* initiative and the new priorities of teacher training bring the geopolitical story full circle. EU funding was obtained to develop the new standard curriculum, and international experts were consulted, in order that the Latvian education system be aligned with those in other EU countries.
